# Onset of exposure to workplace bullying and incident treatment with psychotropic medication – an emulated target trial with 25 309 Swedish and Danish employees

**DOI:** 10.1017/S2045796025100413

**Published:** 2026-01-05

**Authors:** Rebecka Holmgren, Jeppe Karl Sørensen, Reiner Rugulies, Tianwei Xu, Louise Dalsager, Ida E. H. Madsen, Linda L. Magnusson Hanson

**Affiliations:** 1 Stress Research Institute, Division of Psychobiology and Epidemiology, Department of Psychology, Stockholm University, Stockholm, Sweden; 2 Department of Public Health Sciences, Stockholm University, Stockholm, Sweden; 3 National Research Centre for the Working Environment, Copenhagen, Denmark; 4 Section of Epidemiology, Department of Public Health, University of Copenhagen, Copenhagen, Denmark; 5 The National Institute of Public Health, University of Southern Denmark, Copenhagen, Denmark

**Keywords:** antidepressants, common mental disorders, depression, occupational psychiatry, prospective study, violence

## Abstract

**Aims:**

Exposure to workplace bullying is associated with an increased risk of mental health conditions, yet it is debated whether the association is causal. This study aims to address this by examining whether onset of workplace bullying is associated with initiating treatment with psychotropic medication, here used as a proxy measure for onset of common mental disorders.

**Methods:**

We used two longitudinal datasets from Sweden and Denmark (mean age: 47.4, women: 52.8%), combined with national registry data on psychotropic medication purchases. Using a target trial approach, the study population (*N* = 25 309) consisted of employees free of workplace bullying and psychotropic medication use at baseline. We used Cox proportional hazards regression (adjusted for sociodemographic variables, depressive symptoms and psychosocial work characteristics) to assess the association between onset of exposure to workplace bullying and incident treatment with psychotropic medication during 2 years.

**Results:**

In total, 1490 individuals (5.9%) experienced onset of workplace bullying. Bullying onset was associated with incident treatment with any psychotropic medication (HR: 1.42, 95% CI 1.15–1.77, model adjusted for sociodemographic variables). This association was attenuated in the fully adjusted model (HR: 1.24, 95% CI 0.99–1.53). In analyses focusing on antidepressant treatment, the estimates were stronger (HR: 1.55, 95% CI: 1.15–2.09, fully adjusted model). The results further demonstrated an exposure–response relationship, such that higher frequency of bullying exposure was associated with an increased risk of initiating any psychotropic treatment and antidepressants.

**Conclusions:**

Individuals experiencing onset of workplace bullying were at higher risk of starting antidepressant treatment within 2 years. This is the first study showing that onset of workplace bullying can contribute to the development of mental health conditions requiring medical treatment. These results underline the importance of preventive interventions that reduce workplace bullying.

## Introduction

Common mental disorders are a pressing problem in today’s workforce, as demonstrated by increasing rates of mental health-related sickness absences (Blomgren and Perhoniemi, 2022; Dewa *et al.*, 2014) and increased use of psychotropic medication in the adult population worldwide (Brauer *et al.*, 2021). A substantial proportion of the non-fatal health loss globally is specifically attributable to depression and anxiety (World Health Organization, 2017). In addition to the individual burden caused by these disorders, they impose significant societal costs. Thus, it is imperative to identify risk factors for common mental disorders. Mental disorders likely result from multiple and intertwined psychological, biological, social and environmental factors (Kohler *et al.*, 2018; Arango *et al.*, 2021). Today, an increasing body of evidence suggests that the workplace, particularly factors within the psychosocial working environment, play an important role in contributing to decreased mental health among workers (Rugulies *et al.*, 2023).

One notable psychosocial working condition, recently estimated to affect 16% of all workers (Dhanani *et al.*, 2021), is exposure to workplace bullying, defined as repeated and persistent exposure to negative social acts, which involves power imbalance (Einarsen *et al.*, 2011). In a recent meta-analysis of prospective studies, Mikkelsen et al. reported that exposure to workplace bullying was associated with a more than two times higher risk of incident depressive disorder, by far the strongest relative risk of all examined working conditions (Mikkelsen *et al.*, 2021). Another meta-analysis by Verkuil *et al.* (2015) reported prospective associations between exposure to workplace bullying and increased risk of self-reported poor mental health. Prospective studies have further linked exposure to workplace bullying to other indicators of mental health conditions, such as suicidal behaviour (Magnusson Hanson *et al.*, 2023), mental health-related sickness absence (Holmgren *et al.*, 2024) and psychotropic medication use (Conway *et al.*, 2025).

While the existing evidence linking workplace bullying to mental disorders seems to be consistent (Mikkelsen *et al.*, 2021), several methodological concerns limit conclusions on the magnitude of the associations and on causality. First, although the link between bullying and mental health conditions might be bidirectional (Verkuil *et al.*, 2015), only few studies have addressed reverse causation by e.g. adjusting for baseline levels of mental health, focusing on incident outcomes, and/or considering onset or changes of exposure (i.e. to not measure exposure at a random time point). Second, given the presence of residual confounding, none of the previous studies have applied bias analysis techniques to quantify its potential impact on the results. Lastly, some studies rely on self-reported data on both exposure and outcome, increasing the risk of common method bias (Flegal *et al.*, 2018).

One way to overcome the limitations of previous studies would be through a randomized controlled trial in which workers, currently not bullied and with no ongoing or recent mental health conditions, would be randomly assigned to the exposure (workplace bullying) or a control group (remain unexposed to bullying). Ethically, this is unjustifiable. In recent years, however, using observational data for target trial emulations (i.e. mimicking a trial) has been put forward as one way of advancing causal inference in epidemiology (Hernán and Robins, 2016), including research on working conditions and health (Rugulies *et al.*, 2023). Establishing stronger evidence for causality may motivate and help prioritize preventive measures and inform workers’ compensation practices (Madsen & Rugulies, (2021)) as well as provide information on how psychotropic medication use might change under scenarios where workplace bullying is effectively prevented.

### Aim

This study seeks to address the limitations of previous works by using the target trial emulation for examining if onset of exposure to workplace bullying increases the 2-year risk of incident common mental disorders (ascertained by treatment with psychotropic medication) in a multi-cohort study of more than 25 000 employees from two Scandinavian countries.

## Methods

### Study design and sample

We used observational data from two Nordic cohort studies (the Swedish Longitudinal Occupational Survey of Health [SLOSH] and the Work Environment and Health in Denmark Survey [WEHD]), comprising data from biennial surveys concerning work, working conditions and health, with linkages to several national registers via the respondents’ personal identification number. SLOSH, initiated in 2006 with ongoing data collection (comprising 57 104 unique individuals so far), is based on respondents to the population-based Swedish Work Environment Survey in the years 2003–2019. WEHD (comprising 88 076 unique respondents) was initiated in 2012 (data collection ended in 2018), and directed towards a random national sample of Danish citizens, aged 16–64, with a registered income of at least 3000 Danish crones per month at the time of data collection. Detailed descriptions of the cohorts have been published elsewhere (Magnusson Hanson *et al.*, 2018; Sørensen *et al.*, 2025).

Pairing survey data from 2012 to 2018 (response rates 48.2–56.7% in SLOSH and 48.2–56.3% in WEHD) with national registry data, we organized the data into ‘trials’, each spanning two consecutive survey waves (T1 and T2), followed by a 2-year outcome assessment period. Five trials were created: three in SLOSH (T1: 2012, 2014 or 2016; T2: 2014, 2016 or 2018) and two in WEHD (T1: 2012 or 2016; T2: 2014 or 2018; see Supplementary figure 1). Each trial thus required the respondents to have participated in at least two consecutive survey years, and to be in work at both occasions. Eligibility was established using information from surveys and register data. Participants were included if they met the following criteria: (1) aged 16 years or older at T1; (2) unexposed to workplace bullying at T1; (3) no prescription of psychotropic medication during the 3 years preceding T2. Participants were also required to have complete data on all covariates of interest. Individuals who fulfilled eligibility criteria at multiple time points (due to repeated participation in SLOSH/WEHD) were assigned to the first possible trial and thus only included once. Table 1 outlines the full protocol for the target trial emulation.

**Table 1.** Specifications of the ideal and emulated target trial

Causal question of interest	Does onset of exposure to workplace bullying increase the 2-year risk of incident treatment with psychotropic medication?	
	Ideal target trial protocol	Target trial emulation protocol
Eligibility criteria	≥16 years	Participation in two consecutive survey waves (T1 and T2)
	In work	≥16 years at T1
	No recent history of exposure to workplace bullying	In work at T1 and T2
	No ongoing or recent use of psychotropic medications	Unexposed to workplace bullying at T1
		No treatment with psychotropic medication in the 3 years preceding T2
		Not already enrolled in a trial
Treatment strategies	Onset of exposure to workplace bullying vs. remain unexposed to workplace bullying	Same as in target trial.
Treatment assignment	At baseline, through randomization.	Based on reported exposure status in surveys at T2. Respondents are assumed to be comparable after adjustment for baseline covariates.
Follow-up	Starts at the time of treatment initiation, ends after 24 months or in case of incident treatment, emigration or death.	Starts at the time of exposure ascertainment (T2), ends at same time points as target trial, ascertained through national registers.
Primary outcome	Incident treatment with any psychotropic medication (N05B, N05C, N06A).	Same as in target trial.
Secondary outcome	Incident treatment with antidepressants (N06A).	Same as in target trial.
Effects of interest	Hazard ratio (HR) with 95% confidence interval (CI)	Crude and adjusted HRs with 95% CI
Statistical analysis	Cox proportional hazards regression	Cox proportional hazards regression, adjusting for baseline covariates.

The final study sample consisted of 25 309 participants (9480 from SLOSH, 15 829 from WEHD) (supplementary figure 2). In both cohorts, neither missing values on workplace bullying (at T1 or T2), nor on other covariates (at T1) were associated with subsequent psychotropic medication use. Missing values on covariates were not associated with exposure onset in WEHD. In SLOSH, however, a slightly higher proportion of those excluded due to missing values on baseline covariates were subjected to later onset of workplace bullying (11.2% versus 7.9%). Prior studies using data from the same years have reported that, compared with participants responding to one survey only, repeated participation in SLOSH/WEHD was associated with being female, being older and having higher educational attainment, but not related to exposure to workplace bullying (Magnusson Hanson *et al.*, 2018; Holmgren *et al.*, 2023).

### Variables

*Exposure: Onset of workplace bullying.* Workplace bullying was measured using a self-labelling approach (supplementary Table 1), with items asking about frequency of exposure to personal persecution (SLOSH) or bullying (WEHD) from colleagues or supervisors during the past 6 months in SLOSH and 12 months in WEHD. Individuals with affirmative answers were classified as exposed, and those responding ‘never’ were classified as unexposed. We also created a categorical variable indicating exposure frequency (never/sometimes/monthly or more often), to use in exposure-response analyses. Data from T1 were used to establish baseline eligibility (ensuring all participants were unexposed), and data from T2 were used to assess onset of exposure.

*Outcome: Incident treatment with psychotropic medication/antidepressants.* We used data from the Swedish National Prescribed Drug Register (established 2005) and the Danish National Prescription Register (established 1994) containing information on all redeemed medications dispensed at pharmacies in Sweden or Denmark respectively, including date of purchase and product type, classified according to the Anatomical Therapeutic Chemical Classification system (Wettermark *et al.*, 2007; Thygesen *et al.*, 2011). Incident treatment with any psychotropic medication was defined as the first registered purchase of anxiolytics (N05B), sedatives (N05C) or antidepressants (N06A) during a maximum of 24 months of follow-up from T2. Incident treatment with antidepressants was defined as the first registered purchase of N06A during follow-up.

*Covariates.* Based on previous research, we constructed a directed acyclic graph to identify potential confounders (supplementary figure 3). Accordingly, we retrieved data on sex (male/female), age (in years), marital status (single/married or cohabiting), living with children under 18 years (yes/no), educational attainment (low/medium/high, corresponding to elementary or secondary education, <3 years of post-secondary education, and ≥3 years of secondary education, respectively). These variables were retrieved from national registers in Sweden and Denmark at the time point of the first survey participation (T1). We further retrieved survey data on depressive symptoms at T1 (using the Symptom Checklist-core depression scale (SCL-CD6) in SLOSH and the Major Depression Inventory (MDI) in WEHD) (Bech *et al.*, 2001; Magnusson Hanson *et al.*, 2014), job demands, and decision authority at T1 and, for sensitivity analysis on SLOSH participants, job change between T1 and T2 (supplementary table 1).

### Statistical analyses

Descriptive statistics (means and standard deviations for continuous variables, frequencies and percentages for categorical variables) were computed. Smoothed Kaplan–Meier survival curves were generated to illustrate the cumulative incidence of psychotropic medication use over time, stratified by exposure status at T2.

We used Cox proportional hazards models to estimate the association between onset of exposure to workplace bullying and incident treatment with psychotropic medication, using time since survey (in months) as the underlying time scale. Participants were followed for a maximum of 24 months, until incident purchase, death or emigration (death date retrieved from the Swedish National Cause of Death Register/Statistics Denmark’s Cause of Death Register, emigration retrieved from the Swedish LISA-register and Danish Civil Registration System), whatever occurred first. The 24-month follow-up period was chosen because most participants had available follow-up data for this duration. An exception was made for participants in trial 5 (WEHD), for whom the follow-up period ended on 31 December 2019 due to lack of updated register data after that point. We tested the proportional hazard assumptions by visual inspection of the log–log plot, via formal tests of Schoenfeld residuals, and by including time-dependent covariates in our models. Non-proportionality was in general not of any concern (supplementary text 1). Several models were estimated, with increasing levels of adjustment: a crude model; Model 1 (adjusted for sex, age, marital status, cohabiting with children, education, and baseline year); Model 2 (additionally adjusted for T1 job demands and decision authority); and Model 3 (additionally adjusted for T1 depressive symptoms). Due to legal regulations, individual-level data could not be shared between Sweden and Denmark. We pooled the trials within each country and estimated associations in each cohort. Cohort-specific estimations were then pooled using fixed-effects meta-analysis, chosen due to the low number of cohorts (Hedges and Vevea, 1998). Prior studies have demonstrated low heterogeneity between the cohorts (Holmgren *et al.*, 2023). Results are presented as crude and adjusted hazard ratios (HR) with 95% confidence intervals (CI). The analytical process was repeated using incident treatment with antidepressants as the outcome. We further examined exposure-response associations between frequency of workplace bullying and any psychotropic medication treatment, and antidepressant treatment, respectively.

Several sensitivity analyses were performed. The main analysis was stratified by sex (due to low number of cases, these analyses were only performed using any psychotropic medication as the outcome). We assessed how robust our results were to residual confounding by estimating E-values, representing the strength that any unmeasured confounder(s) need to have with both the exposure and the outcome to fully explain away the given association (Vanderweele and Ding, 2017). To assess the influence of time-varying work-related factors, we stratified the main analysis by job change between T1 and T2 (due to data availability this analysis was only conducted in SLOSH, using any psychotropic medication as the outcome). To account for possible bias due to reverse causation we used established cut-offs for SCL-CD6 (≥17)/MDI (≥20) to exclude participants with clinical levels of depression at T1 (205 and 688 individuals in SLOSH and WEHD, respectively), and repeated the main analysis. Lastly, to include possible short-term associations between onset of workplace bullying and incident treatment, we conducted a sensitivity analysis in which the follow-up period was shifted 6 months earlier in SLOSH and 12 months earlier in WEHD, thereby ensuring that the 24-month follow-up fully covered the exposure assessment window (supplementary figure 4). This analysis was included to examine whether immediate treatment responses occurring shortly after bullying onset could influence our main findings. Note that the temporal ordering between exposure and outcome is less clear in these analyses.

Stata (version 18) was used for all cohort-specific analyses. The meta-analysis was performed using the package ‘metafor’ in R Studio (version 4.3.2).

### Ethics statement

Ethical approval has been obtained for SLOSH from the Regional Ethical Review Board in Stockholm, and WEHD has been registered by the Danish Data Protection Agency. Invited participants in SLOSH and WEHD have received written information about the purpose of the questionnaire, that their participation is voluntary, and that they have the right to withdraw from participation at any time.

## Results

During the 2-year period between T1 and T2, 1490 individuals (5.9%) became exposed to workplace bullying (501 [5.3%] and 989 [6.2%] individuals in SLOSH and WEHD, respectively). Among the 1490 exposed individuals, 1119 (75.1%) reported that bullying had occurred ‘sometimes’, whereas 371 (24.9%) reported that it had occurred at least ‘monthly’. Table 2 displays key characteristics at T1, stratified by exposure status at T2. Details for SLOSH, WEHD and each trial can be found in supplementary tables 2a and 2b. Respondents who became exposed to workplace bullying at T2 were at T1 more likely to be female, single, and have lower education, higher depressive symptom scores, higher job demands, and lower decision authority compared to unexposed respondents.

**Table 2.** Characteristics of the study samples at T1, stratified by exposure status at T2

	Total	Unexposed at T2	Exposed at T2
	*N* = 25309	*N* = 23819	*N* = 1490
Sex			
Male	11946 (47.2)	11357 (47.7)	589 (39.5)
Female	13363 (52.8)	12462 (52.3)	901 (60.5)
Age (in years)	47.4 (10.1)	47.5 (10.1)	47.3 (9.8)
Marital status			
Single	4891 (19.3)	4518 (19.0)	373 (25.0)
Married/cohabiting	20418 (80.7)	19301 (81.0)	1117 (75.0)
Children living at home			
No	13284 (52.5)	12477 (52.4)	807 (54.2)
Yes	12025 (47.5)	11342 (47.6)	683 (45.8)
Education			
Low	13191 (52.1)	12338 (51.8)	853 (57.2)
Intermediate	5214 (20.6)	4937 (20.7)	277 (18.6)
High	6904 (27.3)	6544 (27.5)	360 (24.2)
Depressive symptom score^a^	SLOSH: 4.3 (4.4)	SLOSH: 4.2 (4.3)	SLOSH: 5.8 (5.0)
	WEHD: 7.0 (6.3)	WEHD: 6.8 (6.2)	WEHD: 9.8 (7.5)
Job demands score^b^	8.4 (1.7)	8.3 (1.7)	8.9 (1.6)
Decision authority score^c^	7.1 (1.1)	7.1 (1.1)	6.9 (1.2)

Presented as *N* (%) or mean (SD). SLOSH, Swedish Longitudinal Occupational Survey of Health; WEHD,  Working Environment and Health in Denmark study.

a Range 0–24 (SLOSH)/0–50 (WEHD). Since different scales were used, cohort-specific means and standard deviations are displayed.

b Range 3–12.

c Range 2–8.

### Onset of workplace bullying and incident treatment with psychotropic medication

During the 2-year follow-up after T2, we identified 1084 incident treatments with psychotropic medications (SLOSH: 489 cases [5.2%], 26.6 cases per 1000 person-years, mean follow-up time: 23.3 months; WEHD: 595 cases [3.8%], 19.5 cases per 1000 person-years, mean-follow-up time: 23.1 months). When we restricted the analyses to incident antidepressant treatment, we identified 465 cases (SLOSH: 219 cases, 11.9 cases per 1000 person-years, mean follow-up time: 23.7 months; WEHD: 246 cases, 8.1 cases per 1000 person-years, mean follow-up time: 23.4 months). Smoothed Kaplan-Meier survival curves are presented in supplementary figures 5a, 5b, 6a and 6b.

**Figure 1.**
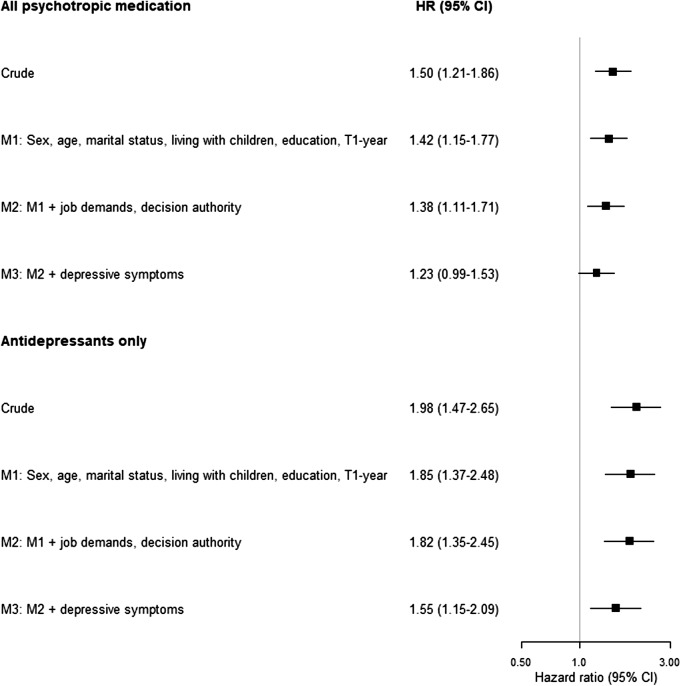
Association between workplace bullying and incident treatment with psychotropic medication.

Onset of exposure to workplace bullying was associated with an increased risk of incident treatment with any psychotropic medication (pooled HR_M1_: 1.42, 95% CI: 1.15–1.77), after adjustment for sex, age, marital status, children living at home and baseline year (Figure 1 and supplementary table 4). When further adjusting for depressive symptoms and psychosocial work characteristics at T1, the point estimate was attenuated with the lower confidence limit including unity (pooled HR_M3_: 1.23, 95% CI: 0.99–1.53). We observed an exposure-response relationship between frequency of bullying and incident treatment with any psychotropic medication (*p*_trend_ = 0.031), with a pooled HR_M3_ of 1.47 (95% CI: 1.00–2.17) for employees with exposure occurring at least monthly (supplementary table 5).

When we restricted the analyses to incident treatment with antidepressants, onset of exposure to workplace bullying was associated with an increased risk in all models, including the most-adjusted model (pooled HR_M3_: 1.55, 95% CI: 1.15–2.09; Figure 1 and supplementary table 4), with an exposure-response relationship (p_trend_ = 0.0009). Comparing with those unexposed, there was a more than two times higher risk of incident treatment with antidepressants for employees experiencing at least monthly exposure (pooled HR_M3_:2.10, 95% CI: 1.30–3.40) (supplementary table 5).

E-value calculations indicated that any unmeasured confounder(s) would need to increase the risk of both workplace bullying onset and psychotropic treatment initiation by 1.6 times, or antidepressant treatment initiation by 2.1 times, to nullify these results (supplementary table 6).

### Subgroup analyses

In the subgroup analyses, we stratified by sex (women, men), cohort (SLOSH, WEHD) and employment in the same organization from T1 to T2 (yes/no) as well as restricted the sample to individuals without presence of elevated depressive symptoms at T1. Hazard ratios were similar to the estimates in the main analyses (supplementary figure 7).

### Onset of workplace bullying and incident treatment with psychotropic medication when including cases that occurred between T1 and T2

As a final sensitivity analysis, we examined the association between onset of workplace bullying and incident treatment with psychotropic medication while also including cases that occurred between T1 and T2. Sample characteristics were similar to those of the main sample (see supplementary figure 8 for the sampling process and supplementary table 7 for sample characteristics). In the most-adjusted model, the estimates were slightly stronger for all psychotropic medications (HR_M3_: 1.35, 95% CI: 1.12–1.64) and slightly weaker for antidepressants (HR_M3_: 1.42, 95% CI: 1.05–1.93), but overall similar to the estimates in the main analysis (supplementary figure 9 and supplementary table 8).

## Discussion

In this target trial emulation, using pooled data from two large Nordic cohorts of working-aged individuals, we found that employees without current or recent psychotropic treatment, who experienced onset of workplace bullying had a 1.2 times higher risk of initiating treatment with any type of psychotropic medication and a 1.6 times higher risk of initiating antidepressant treatment within the next 2 years, compared to unexposed employees. There was an exposure-response relationship between increasing frequency of bullying and increased risk of both any psychotropic treatment and treatment with antidepressants.

To our knowledge, this is the first study to show that new episodes of workplace bullying can contribute to the development of adverse mental health conditions, requiring medical treatment. Guided by the target trial framework, we used observational data to emulate a series of target trials to address concerns in the literature that previously reported associations between workplace bullying and risk of mental disorders may have been biased by undetected depressive symptoms at baseline. Neither adjusting for baseline depressive symptoms nor restricting the analyses to workers without clinical levels of depressive symptoms at baseline did significantly alter the risk estimate, increasing our confidence that the estimates in our analyses indicate a causal relationship between workplace bullying and common mental disorders. The associations were consistent in Sweden and Denmark and remained similar when the follow-up period was adjusted to include potential immediate treatment initiations.

### Comparison with previous studies

Direct comparisons with prior studies are difficult, as we are not aware of any research examining onset of workplace bullying in relation to subsequent psychotropic drug use or confirmed mental disorders. However, our findings align with meta-analytic evidence of a prospective association between exposure to workplace bullying and depressive episodes (Mikkelsen *et al.*, 2021), and with two previous prospective studies, which found that exposure to workplace bullying (measured at one point in time) was associated with an increased risk of psychotropic medication (Lallukka *et al.*, 2012; Conway *et al.*, 2025). Prior cross-sectional research on workplace bullying and self-reported psychotropic medication has shown mixed findings regarding exposure-response associations (Vartia, 2001; Niedhammer *et al.*, 2011) and we are not aware of previous prospective studies that have analysed exposure-response associations.

### Strengths and limitations

The main strength of the study is the use of observational data to emulate a series of target trials. We used transparent and strict inclusion criteria and temporal sequencing of exposure and outcome to reduce risks of bias. Our data allowed a longitudinal study design, including repeated measures of the exposure, in a large study sample of 25 309 employees.

These strengths are balanced by several limitations. Exposure to workplace bullying was assessed through self-report, which may be influenced by individual differences in perception and potential changes in sensitivity to interpersonal behaviours over time (Notelaers and Van Der Heijden, 2021). Although we excluded individuals with ongoing or recent psychotropic medication use and adjusted for baseline depressive symptoms to reduce this risk, time-varying reporting bias cannot be entirely ruled out. Moreover, the specific measures of workplace bullying differed between SLOSH and WEHD, which is why we display both cohort-specific and pooled estimates. In addition, both here and in previous works (Xu *et al.*, 2020; Holmgren *et al.*, 2023), the overall associations between bullying exposure and health outcomes have been similar using these two measures, suggesting that the same phenomenon is being assessed across the two cohorts. Exposure to workplace bullying was assessed at 2-year intervals, requiring us to assume that reported bullying reflected exposure status during the entire eligibility period, potentially leading to misclassification of exposure onset. In addition, bullying onset was reported retrospectively for a 6- or 12-month period without precise dates, preventing alignment of the start of follow-up with the exact timing of exposure onset. We may thus have excluded early psychotropic treatment cases and underestimated the associations in our main models. Notably, the sensitivity analyses in which the follow-up window was shifted backwards showed largely similar estimates, indicating that such early treatment cases are unlikely to have substantially biased the main results.

The use of a registry-based outcome (purchase of psychotropic medication) reduces the risk of common method bias and loss-to-follow-up. However, psychotropic medications are not exclusively prescribed for mental disorders, and not all individuals with mental disorders receive or adhere to medical treatment (Thielen *et al.*, 2009; Weye *et al.*, 2023). Therefore, outcome misclassification might have occurred, potentially leading to an underestimation of the results. Although our sample size was considerably large, the statistical power was still limited for performing analyses on other types of psychotropic medications than antidepressants (e.g., hypnotics and sedatives).

We tried to address the impact of unmeasured confounding, e.g., from genetics and familial factors, adverse childhood experiences and/or personality traits (Anda *et al.*, 2007; Jacobsen *et al.*, 2018; Kizuki *et al.*, 2020; Ormel *et al.*, 2013; Nielsen and Knardahl, 2015), by calculating E-values. Our E-value calculations showed that the analysis of antidepressant medication was more robust to unmeasured confounding than the analysis of all types of psychotropic medications While unmeasured confounding cannot be ruled out, we consider the risk of it substantially influencing the results to be relatively low, especially given our eligibility criteria, which only included individuals who had not previously been exposed to workplace bullying.

Selection bias may exist as our eligibility criteria required repeated survey participation. However, we have reduced this risk by adjusting for covariates associated with repeated participation in cohort waves. Moreover, as workplace bullying itself is not likely to contribute to the selection mechanism (Magnusson Hanson *et al.*, 2018; Holmgren *et al.*, 2023), we consider the risk of selection bias to be limited. We only used cohorts from two Scandinavian countries. Therefore, caution should be paid when generalizing our findings to a vastly different context.

## Conclusions

This emulated target trial showed that onset of workplace bullying is associated with an increased risk of incident common mental disorders, as indicated by the initiation of psychotropic treatment. Specifically, individuals reporting onset of workplace bullying were at higher risk of starting antidepressant treatment within 2 years following exposure. Under the assumption that this association is causal, the results underscore the importance of preventive policies and interventions that reduce workplace bullying, and mitigate its adverse outcomes.

## Supporting information

10.1017/S2045796025100413.sm001 Holmgren et al. supplementary material Holmgren et al. supplementary material

## Data Availability

The SLOSH data generated and analyzed during the current study is not publicly available due to ethical restrictions and considering that sensitive personal data are involved. Access to the data may be provided to other researchers in line with Swedish law and after consultation with the Stockholm University legal department. Requests for SLOSH data, stored at the Department of Psychology, Stockholm University should be sent to data@slosh.se. WEHD is based on anonymized microdata available from Statistics Denmark. Access to data can only be permitted through an affiliation with a Danish authorized environment.

## Supplementary material

The supplementary material for this article can be found at https://doi.org/10.1017/S2045796025100413.
