# Supplementary material for: Onset of exposure to workplace bullying and incident treatment with psychotropic medication – an emulated target trial with 25 309 Swedish and Danish employees
Source: Epidemiol Psychiatr Sci. 2026 Jan 5;35:e3. doi: 10.1017/S2045796025100413 (PMC12816931; doi:10.1017/S2045796025100413)
Supplement: Holmgren et al. supplementary material [file S2045796025100413sup001.pdf]

Supplementary material

Supplement to: **Onset of exposure to workplace bullying and incident treatment with psychotropic medication – an emulated target trial with 25 309 Swedish and Danish employees**

Holmgren, R., Sørensen, J.K., Rugulies, R, Xu, T., Dalsager, L., Madsen, I.E.H., Magnusson Hanson, L.L.

Table of contents

Supplementary figure 1. Overview of study design. 1

Supplementary figure 2. Flowchart of sample selection process. 2

Supplementary table 1. Overview of survey-based covariates. 3

Supplementary figure 3. Directed acyclic graph. 5

Supplementary text 1. Proportional hazards assumption. 6

Supplementary figure 4. Overview of study design in sensitivity analysis with earlier start of follow-up period. 7

Supplementary table 2a. Characteristics of the study samples at T1, stratified by cohort and exposure status at T2. 8

Supplementary table 2b. Characteristics of the study samples, stratified by trial. 9

Supplementary figures 5a-b. Smoothed Kaplan-Meier survival curves of treatment with psychotropic medication in SLOSH and WEHD. 10

Supplementary figures 6a-b. Smoothed Kaplan-Meier survival curves of treatment with antidepressants in SLOSH and WEHD. 11

Supplementary table 4. Cohort-specific and pooled estimates from Cox proportional hazards regressions, examining the association between onset of workplace bullying and incident treatment with psychotropic medications/antidepressants during 2 years of follow-up starting at T2. 12

Supplementary table 5. Cohort-specific and pooled estimates from Cox proportional hazards regressions, examining exposure-response association between onset of workplace and incident treatment with psychotropic medication/antidepressants during 2 years of follow-up starting at T2. 13

Supplementary table 6. Estimated E-values. 14

Supplementary figure 7. Subgroup analyses of the association between workplace bullying and incident treatment with psychotropic medication/antidepressants during 2 years of follow-up starting at T2. 15

Supplementary figure 8. Flowchart of sample selection process for sensitivity analyses, starting follow-up 6/12 months earlier. 16

Supplementary table 7. Characteristics of the study samples used for sensitivity analysis, starting follow-up 6/12 months earlier. 17

Supplementary figure 9. Association between workplace bullying and incident treatment with psychotropic medication including cases that occurred between T1 and T2 18

Supplementary table 8. Cohort-specific and pooled estimates from sensitivity analysis using Cox proportional hazards regressions, examining the association between onset of workplace bullying and incident treatment with psychotropic medications/antidepressants during 2 years of follow-up starting at the exposure reference period (T2 minus 6 months in SLOSH, T2 minus 12 months in WEHD). 19

Supplementary figure 1. Overview of study design.

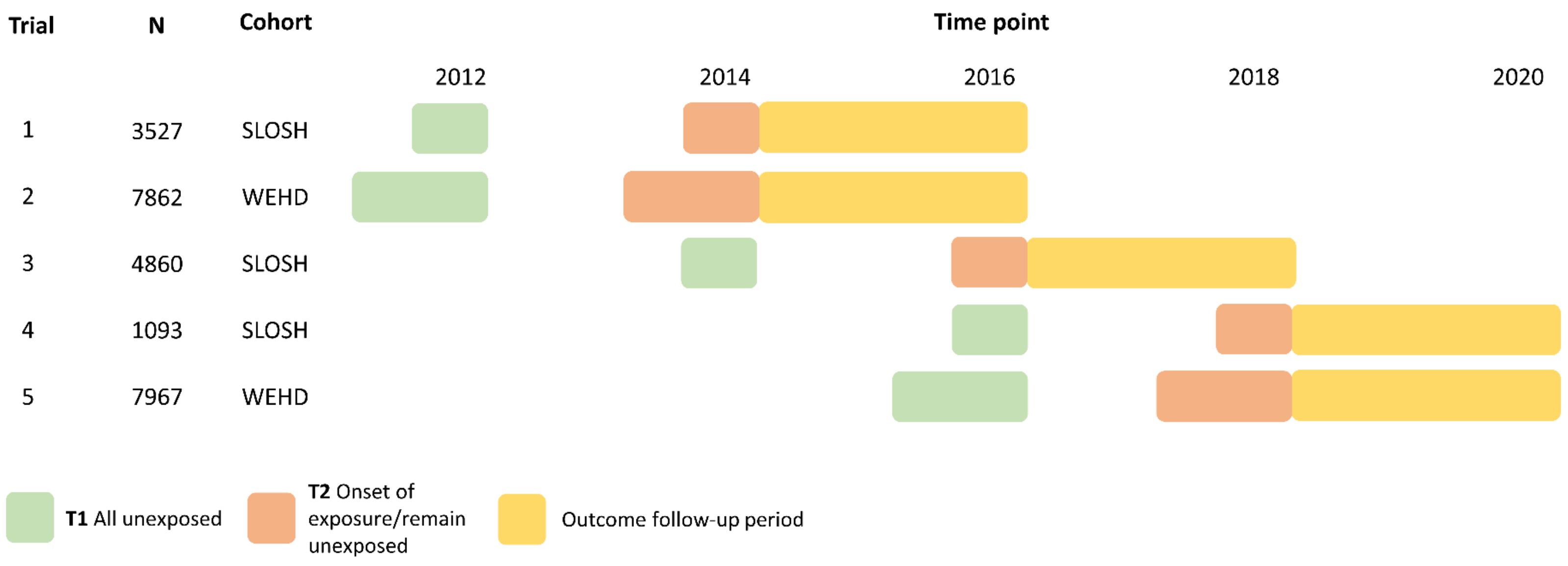

Supplementary figure 2. Flowchart of sample selection process.

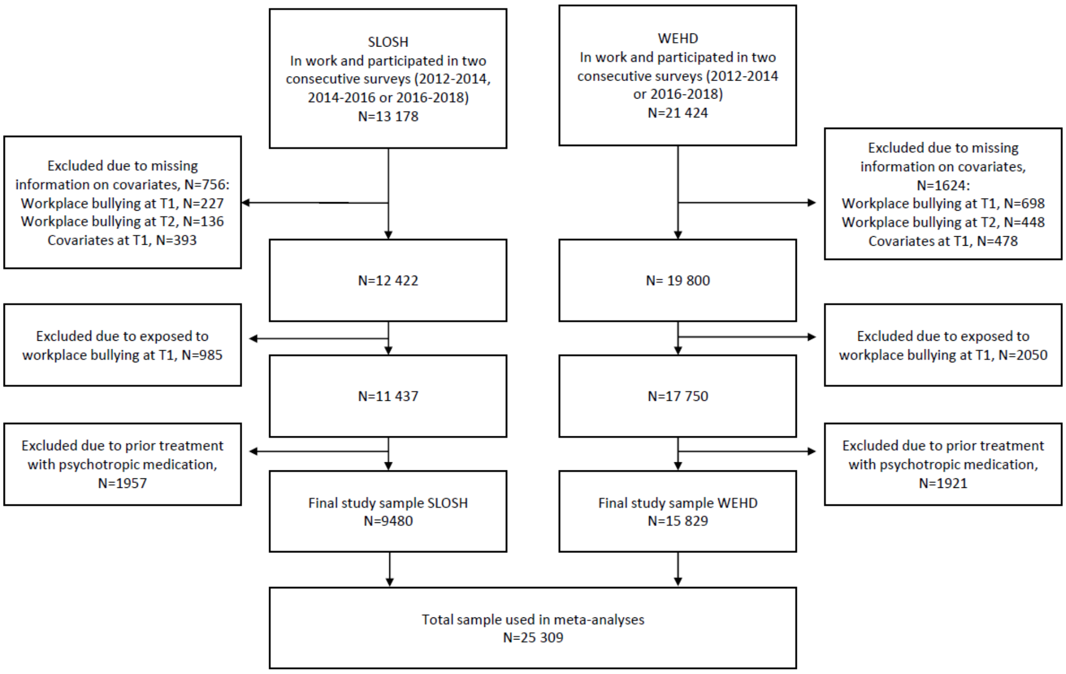

Supplementary table 1. Overview of survey-based covariates.

<table>
<tr><th>Covariate</th><th>Survey</th><th>Survey item(s) and response options</th><th>Coding</th></tr>
<tr><td rowspan="2">Workplace bullying</td><td>SLOSH</td><td>2012-2016:
During the last 6 months, have you been subjected to personal persecution in the form of unkind words or behaviors from superiors or fellow workers?
2018:
During the last 6 months, have you been subjected to personal persecution in the form of unkind words or behaviors from superiors?
During the last 6 months, have you been subjected to personal persecution in the form of unkind words or behaviors from fellow workers?
• Yes, one or several times a week
• Yes, one or several times a month
• Yes, sometime during the last 6 months
• No</td><td>The variable was recoded into a binary variable, with affirmative answers indicating exposure.</td></tr>
<tr><td>WEHD</td><td>Bullying occurs when one or more individuals regularly and over a longer period of time – or repeatedly in a rough way – subjects one or more individual to abusive acts, which are perceived as hurtful or degrading by the targeted individual(s)

Have you been exposed to bullying at work within the past 12 months?
• Yes, daily
• Yes, weekly
• Yes, monthly
• Yes, seldomly
• No, never

If yes, who bullied you? (multiple answers possible)
• Colleagues
• Managers
• Subordinates
• Customers, clients, patients, students etc.</td><td>The variable was recoded into a binary variable, with affirmative answers indicating exposure. Participants who reported exposure to workplace bullying by external sources only were treated as unexposed (T2 N=152). Participants who answered affirmative on exposure to workplace bullying but did not indicate by whom they were exposed were treated as exposed.</td></tr>
<tr><td rowspan="2">Depressive symptoms and depression</td><td>SLOSH</td><td>The Symptom Checklist-core depression scale (SCL-CD6) consisting of 6 items assessing depressive symptoms during the last week.

Example item: How much during the last week have you been bothered by feeling low in energy?

Responses are rated on a scale ranging from 0 (not at all) to 4 (very much)</td><td>A sum score (range 0-24) was calculated for all participants with no missing values on unique items, with higher values indicating higher levels of depressive symptoms.

A sum score of 17 or higher was used to indicate presence of depression.</td></tr>
<tr><td>WEHD</td><td>The Major Depression Inventory (MDI) consisting of 12 items assessing depressive symptoms during the last two weeks.

Example item: How often during the last two weeks have you felt low in spirits or sad?

Responses are rated on a scale ranging from 0 (not at all) to 5 (all the time)</td><td>A sum score (range 0-50) was calculated for all participants with no missing values on unique items, with higher values indicating higher levels of depressive symptoms. According to recommendations, two pairs of items are combined, using only the item with the highest score to calculate the sum score.

A sum score of 20 or higher was used to indicate presence of depression.</td></tr>
<tr><td rowspan="2">Job demands</td><td>SLOSH</td><td>The Demand-Control-Support-Questionnaire:

Do you have to work fast?
Do you have enough time to do everything?
Does your work often involve conflicting demands?

Responses are rated on a scale ranging from: 1 (yes, often) to 4 (no, never)</td><td>2 items were reversed. A sum score (range 3-12) was calculated for all participants with no missing values on unique items, with higher values indicating higher levels of job demands.</td></tr>
<tr><td>WEHD</td><td>How often is it necessary to keep a high work pace?
How often do you have time enough for your work tasks?
How often are contradictory demands placed on you at your work?</td><td>2 items were reversed. 2 response alternatives were collapsed, so that responses on each item ranged from 1-4.</td></tr>
</table>

		Responses are rated on a scale ranging from 1 (always) to 5 (never)	A sum score (range 3-12) was calculated for all participants with no missing values on unique items, with higher values indicating higher levels of job demands.
Decision authority	SLOSH	The Demand-Control-Support-Questionnaire:<br><br>Do you have a choice in deciding how you do your work?<br>Do you have a choice in deciding what you do at work?<br><br>Responses are rated on a scale ranging from: 1 (yes, often) to 4 (no, never)	The items were reversed. A sum score (range 2-8) was calculated for all participants with no missing values on unique items, with higher values indicating higher levels of decision authority.
	WEHD	How often can you influence how you solve your work tasks?<br>How often can you influence when you solve your work tasks?<br><br>Responses are rated on a scale ranging from 1 (always) to 5 (never).	The items were reversed. 2 response alternatives were collapsed, so that responses on each item ranged from 1-4.<br>A sum score (range 2-8) was calculated for all participants with no missing values on unique items, with higher values indicating higher levels of decision authority.
Job change	SLOSH	The following question apply to your place(s) of work in the past two years:<br>Have you changed job?<br>• No<br>• Yes, once<br>• Yes, one time<br>• Yes, 2-3 times<br>• Yes, 4 times or more	The variable was recoded into a binary variable indicating yes (one time or more) or no.

Supplementary figure 3. Directed acyclic graph.

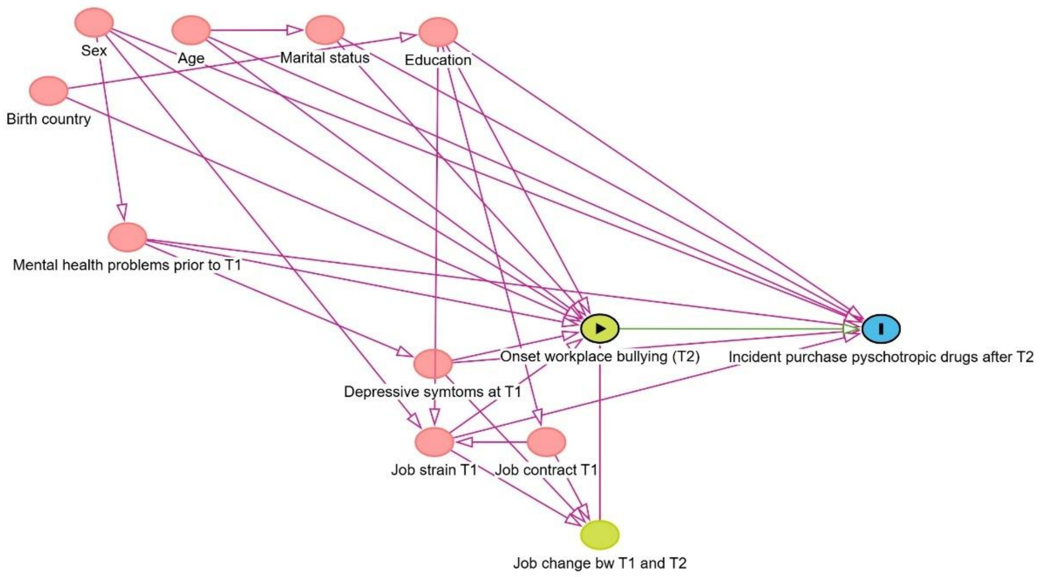

Supplementary text 1. Proportional hazards assumption.

In SLOSH, specifically for the analyses of antidepressant medication, there was some uncertainty regarding whether the proportional hazards assumption was fulfilled or not, most likely having to do with few cases of antidepressant treatment among the exposed. The log-log plot showed crossing lines (at around 9 months of follow-up) indicating violation of the proportional hazards assumption. However, formal tests (using Schoenfeld residuals) and tests of exposure-time-interaction did not indicate that the proportional hazards assumption was violated. We decided to conduct a sensitivity analysis in SLOSH, where we split the follow-up time at 9 months (based on the crossing lines of the log-log-plot) and ran the analyses separately for each time period. In these analyses, the HR for antidepressant medication was 0.8 (95% CI: 0.2-2.4) in the first period (follow-up 0-9 months) and 2.2 (95% CI: 1.3-3.8) in the second period (follow-up from month 9 and onwards). Based on this, we concluded that the HR of 1.67 (1.02-2.74) for the entire follow-up period could be considered a conservative estimate and decided to proceed with analyses as described in the methods section.

Supplementary figure 4. Overview of study design in sensitivity analysis with earlier start of follow-up period.

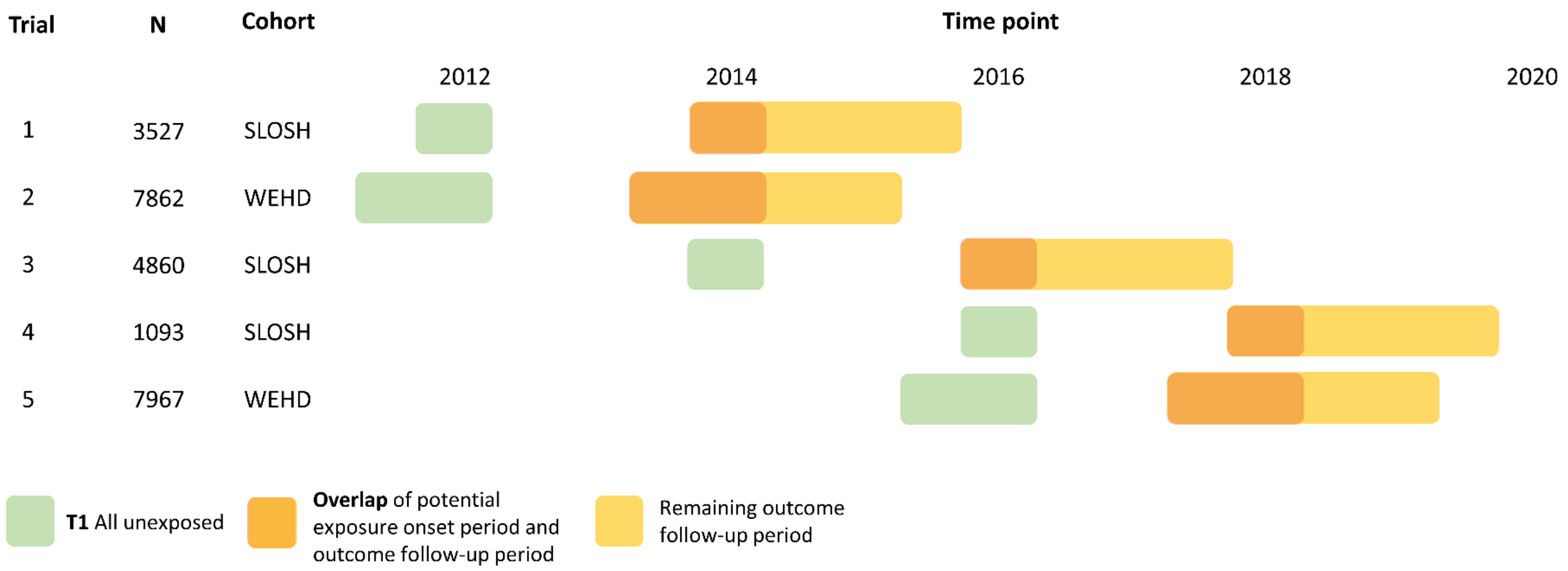

Supplementary table 2a. Characteristics of the study samples at T1, stratified by cohort and exposure status at T2.

	SLOSH			WEHD		
	Total<br>9480	Unexposed at T2<br>8979	Exposed at T2<br>501	Total<br>15829	Unexposed at T2<br>14840	Exposed at T2<br>989
Sex						
Male	4365 (46.0)	4179 (46.5)	186 (37.1)	7581 (47.9)	7178 (48.4)	403 (40.7)
Female	5115 (54.0)	4800 (53.5)	315 (62.9)	8248 (52.1)	7662 (51.6)	586 (59.3)
Age	49.6 (9.6)	49.7 (9.6)	49.0 (9.1)	46.1 (10.4)	46.1 (10.4)	46.5 (10.1)
Marital status						
Single	1793 (18.9)	1669 (18.6)	124 (24.8)	3098 (19.6)	2849 (19.2)	249 (25.2)
Married/cohabiting	7687 (81.1)	7310 (81.4)	377 (75.2)	12731 (80.4)	11991 (80.8)	740 (74.8)
Children living at home						
No	5312 (56.0)	5032 (56.0)	280 (55.9)	7972 (50.4)	7445 (50.2)	527 (53.3)
Yes	4168 (44.0)	3947 (44.0)	221 (44.1)	7857 (49.6)	7395 (49.8)	462 (46.7)
Education						
Low	4434 (46.8)	4191 (46.7)	243 (48.5)	8757 (55.3)	8147 (54.9)	610 (61.7)
Intermediate	725 (7.6)	701 (7.8)	24 (4.8)	4489 (28.4)	4236 (28.5)	253 (25.6)
High	4321 (45.6)	4087 (45.5)	234 (46.7)	2583 (16.3)	2457 (16.6)	126 (12.7)
Depressive symptom score[1]	4.3 (4.4)	4.2 (4.3)	5.8 (5.0)	7.0 (6.3)	6.8 (6.2)	9.8 (7.5)
Job demands score[2]	7.5 (1.7)	7.5 (1.6)	8.1 (1.7)	8.9 (1.7)	8.8 (1.7)	9.3 (1.6)
Decision authority score[3]	6.3 (1.4)	6.3 (1.4)	6.0 (1.4)	7.6 (0.9)	7.6 (0.9)	7.4 (1.0)
Onset workplace bullying						
Yes	501 (5.3)			989 (6.2)		
No	8979 (94.7)			14840 (93.8)		
Frequency workplace bullying						
Never	8979 (94.7)			14840 (93.8)		
Sometimes	425 (4.5)			694 (4.4)		
≥Monthly	76 (0.8)			295 (1.9)		

Presented as N (%) or mean (SD). SLOSH=Swedish Longitudinal Occupational Survey of Health, WEHD=Working Environment and Health in Denmark study.

[1]range 0-24 (SLOSH)/0-50 (WEHD)

[2]range 3-12 in both cohorts

[3]range 2-8 in both cohorts

Supplementary table 2b. Characteristics of the study samples, stratified by trial.

	SLOSH			WEHD	
Baseline year	2012<br>N=3527	2014<br>N=4860	2016<br>N=1093	2012<br>N=7862	2016<br>N=7967
Sex					
Male	1627 (46.1)	2230 (45.9)	508 (46.5)	3787 (48.2)	3794 (47.6)
Female	1900 (53.9)	2630 (54.1)	585 (53.5)	4075 (51.8)	4173 (52.4)
Age	50.2 (9.2)	49.5 (9.7)	48.2 (10.1)	45.8 (10.4)	46.4 (10.4)
Marital status					
Single	675 (19.1)	909 (18.7)	209 (19.1)	1476 (18.8)	1622 (20.4)
Married/cohabiting	2852 (80.9)	3951 (81.3)	884 (80.9)	6386 (81.2)	6345 (79.6)
Children living at home					
No	1966 (55.7)	2765 (56.9)	581 (53.2)	3918 (49.8)	4054 (50.9)
Yes	1561 (44.3)	2095 (43.1)	512 (46.8)	3944 (50.2)	3913 (49.1)
Education					
Low	1762 (50.0)	2208 (45.4)	464 (42.5)	4358 (55.4)	4399 (55.2)
Intermediate	277 (7.9)	369 (7.6)	79 (7.2)	2239 (28.5)	2250 (28.2)
High	1488 (42.5)	2283 (47.0)	550 (50.3)	1265 (16.1)	1318 (16.5)
Depressive symptom score[1]	3.9 (4.2)	4.5 (4.4)	4.6 (4.5)	6.8 (6.2)	7.1 (6.5)
Demands score[2]	7.5 (1.6)	7.5 (1.6)	7.5 (1.7)	8.8 (1.7)	8.9 (1.7)
Decision authority score[3]	6.2 (1.4)	6.3 (1.4)	6.3 (1.4)	7.6 (0.9)	7.6 (0.9)
Onset workplace bullying					
Yes	215 (6.1)	226 (4.7)	60 (5.5)	507 (6.4)	482 (6.0)
No	3312 (93.9)	4634 (95.3)	1033 (94.5)	7355 (93.6)	7485 (94.0)

Presented as N (%) or mean (SD). SLOSH=Swedish Longitudinal Occupational Survey of Health, WEHD=Working Environment and Health in Denmark study.

[1]range 0-24 (SLOSH)/0-50 (WEHD)

[2]range 3-12 in both cohorts

[3]range 2-8 in both cohorts

Supplementary figures 5a-b. Smoothed Kaplan-Meier survival curves of treatment with psychotropic medication in SLOSH and WEHD.

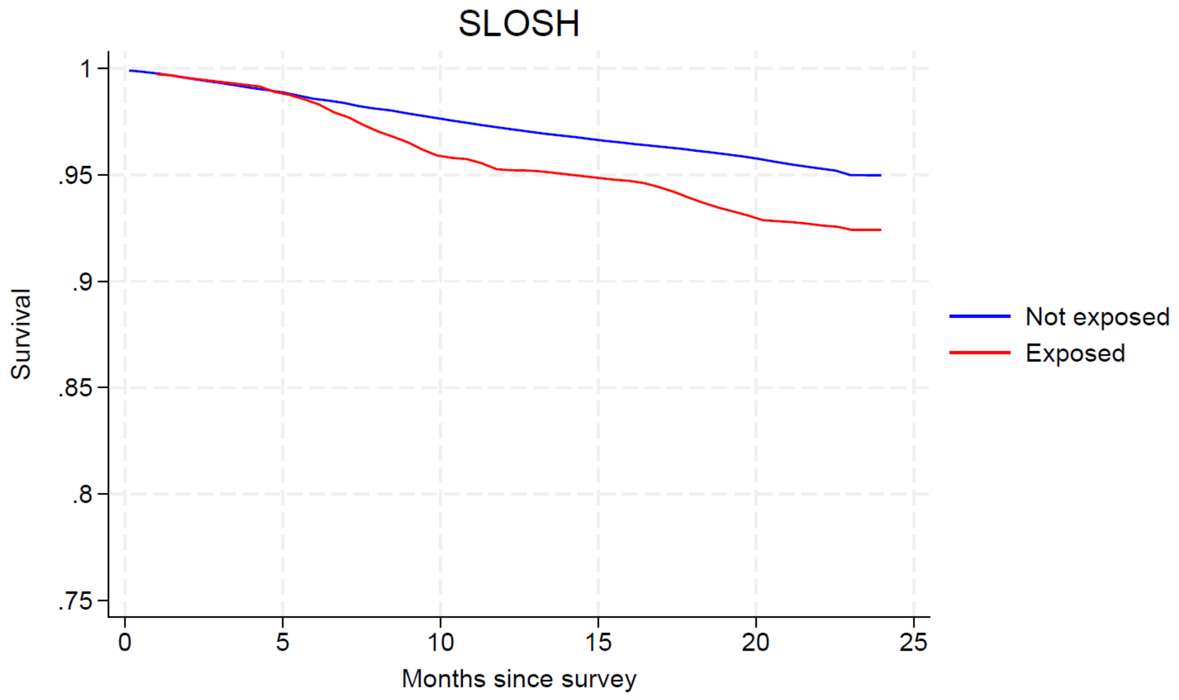

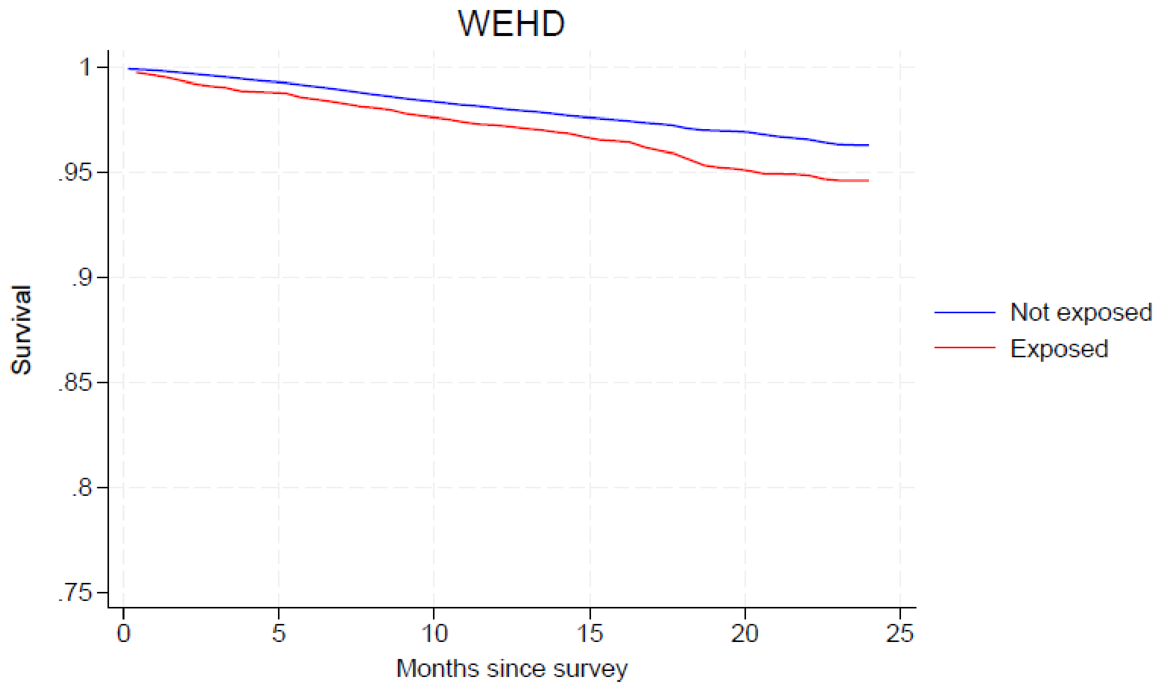

Supplementary figures 6a-b. Smoothed Kaplan-Meier survival curves of treatment with antidepressants in SLOSH and WEHD.

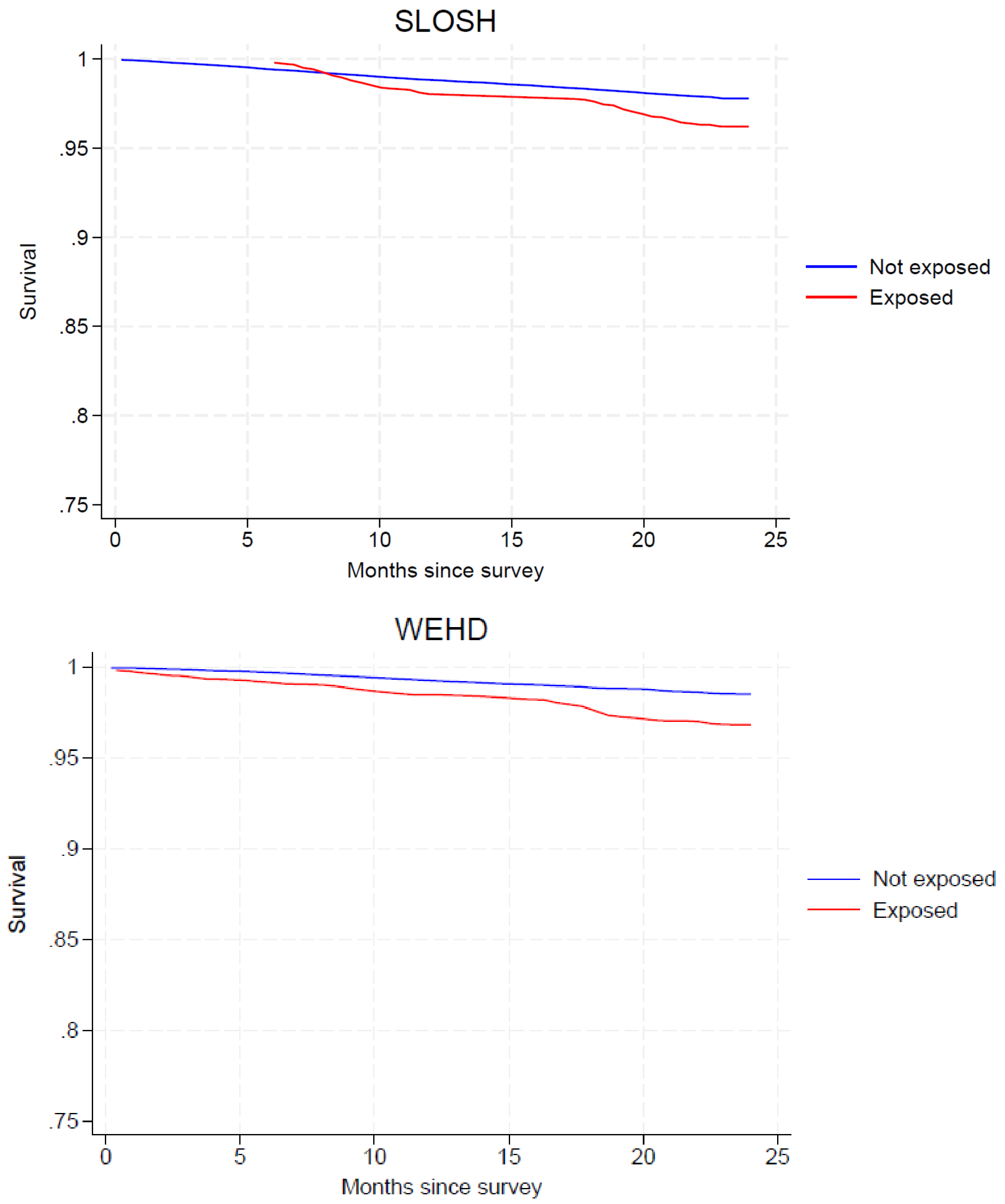

Supplementary table 4. Cohort-specific and pooled estimates from Cox proportional hazards regressions, examining the association between onset of workplace bullying and incident treatment with psychotropic medications/antidepressants during 2 years of follow-up starting at T2.

			SLOSH				WEHD				Pooled estimate
**Outcome**	**Subgroup**	**Model**	**N**	**N exp**	**N cases**	**HR (95% CI)**	**N**	**N exp**	**N cases**	**HR (95% CI)**	**HR (95% CI)**
Psychotropic medication	All	Crude	9480	501	489	1.53 (1.10-2.13)	15829	989	595	1.48 (1.11-1.96)	1.50 (1.21-1.86)
		Model 1				1.44 (1.03-2.00)				1.41 (1.06-1.87)	1.42 (1.15-1.77)
		Model 2				1.38 (0.99-1.93)				1.38 (1.04-1.82)	1.38 (1.11-1.71)
		Model 3				1.28 (0.92-1.79)				1.20 (0.91-1.60)	1.23 (0.99-1.53)
	Women	Crude	5115	315	324	1.53 (1.04-2.24)	8248	586	382	1.40 (1.00-1.97)	1.46 (1.13-1.88)
		Model 1				1.49 (1.02-2.19)				1.40 (1.0-1.97)	1.44 (1.12-1.85)
		Model 2				1.46 (0.99-2.14)				1.35 (0.96-1.90)	1.40 (1.08-1.80)
		Model 3				1.38 (0.94-2.03)				1.17 (0.83-1.65)	1.26 (0.97-1.63)
	Men	Crude	4365	186	165	1.30 (0.66-2.54)	7581	403	213	1.46 (0.88-2.43)	1.40 (0.93-2.10)
		Model 1				1.31 (0.67-2.57)				1.44 (0.86-2.40)	1.39 (0.92-2.09)
		Model 2				1.21(0.61-2.38)				1.44 (0.86-2.40)	1.35 (0.90-2.04)
		Model 3				1.06 (0.54-2.09)				1.29 (0.77-2.16)	1.20 (0.80-1.82)
	Without clinically elevated depressive symptoms at T1	Crude	9275	483	463	1.51 (1.07-2.13)	15141	890	545	1.34 (0.98-1.84)	1.41 (1.12-1.79)
		Model 1				1.41 (1.00-1.99)				1.28 (0.93-1.75)	1.34 (1.06-1.69)
		Model 2				1.36 (0.96-1.93)				1.25 (0.91-1.72)	1.30 (1.03-1.64)
		Model 3				1.26 (0.89-1.79)				1.11 (0.81-1.53)	1.18 (0.93-1.49)
	Employed at same organization between T1 and T2*	Crude	7603	368	392	1.47 (0.99-2.18)	*N.A.*	*N.A.*	*N.A.*	*N.A.*	*N.A.*
		Model 1				1.40 (0.95-2.07)				*N.A.*	*N.A.*
		Model 2				1.33 (0.90-1.97)				*N.A.*	*N.A.*
		Model 3				1.25 (0.85-1.85)				*N.A.*	*N.A.*
	Changed employer between T1 and T2*	Crude	1877	133	97	1.71 (0.91-3.20)	*N.A.*	*N.A.*	*N.A.*	*N.A.*	*N.A.*
		Model 1				1.51 (0.80-2.85)				*N.A.*	*N.A.*
		Model 2				1.56 (0.82-2.96)				*N.A.*	*N.A.*
		Model 3				1.47 (0.77-2.80)				*N.A.*	*N.A.*
Antidepressant medication	All	Crude	9480	501	219	1.71 (1.07-2.74)	15829	989	246	2.17 (1.49-3.17)	1.98 (1.47-2.65)
		Model 1				1.56 (0.97-2.51)				2.05 (1.41-2.99)	1.85 (1.37-2.48)
		Model 2				1.55 (0.96-2.50)				2.01 (1.37-2.94)	1.82 (1.35-2.45)
		Model 3				1.40 (0.87-2.26)				1.66 (1.13-2.43)	1.55 (1.15-2.09)
	Without clinically elevated depressive symptoms at T1	Crude	9275	483	203	1.67 (1.02-2.74)	15141	890	217	2.0 (1.31-3.05)	1.85 (1.34-2.56)
		Model 1				1.53 (0.93-2.52)				1.90 (1.24-2.91)	1.73 (1.25-2.40)
		Model 2				1.53 (0.92-2.52)				1.87 (1.22-2.86)	1.72 (1.24-2.38)
		Model 3				1.39 (0.84-2.30)				1.60 (1.04-2.45)	1.51 (1.09-2.09)

Model 1 adjusted for: sex (not in sex-stratified analyses), age, marital status, cohabiting with children, education and baseline year. Model 2 adjusted for covariates in model 1+T1 job demands and T1 decision authority. Model 3 adjusted for covariates in model 2+T1 depressive symptoms.
*Only performed in SLOSH due to data availability. HR=Hazard rate, CI=confidence interval, N.A.=Not applicable

Supplementary table 5. Cohort-specific and pooled estimates from Cox proportional hazards regressions, examining exposure-response association between onset of workplace and incident treatment with psychotropic medication/antidepressants during 2 years of follow-up starting at T2.

	**Exposure status**	**N**	**Cases**	**Crude HR (95% CI)**	**Model 1 HR (95% CI)**	**Model 2 HR (95% CI)**	**Model 3 HR (95% CI)**
**Psychotropic medication**							
**SLOSH**	Never	8979	451	*Ref*	*Ref*	*Ref*	*Ref*
	Occasionally	425	33	1.57 (1.10.-2.24)	1.49 (1.05-2.13)	1.44 (1.01-2.05)	1.35 (0.95-1.93)
	Frequently	76	5	1.31 (0.54-3.17)	1.16 (0.48-2.80)	1.11 (0.46-2.68)	0.95 (0.39-2.31)
**WEHD**	Never	14840	542	*Ref*	*Ref*	*Ref*	*Ref*
	Occasionally	694	31	1.23 (0.85-1.76)	1.16 (0.85-1.67)	1.14 (0.79-1.64)	1.02 (0.71-1.47)
	Frequently	295	22	2.07 (1.35-3.17)	2.02 (1.31-3.09)	1.95 (1.27-2.99)	1.63 (1.06-2.51)
**Pooled**	Never	23819	993	*Ref*	*Ref*	*Ref*	*Ref*
	Occasionally	1119	64	1.39 (1.08-1.79)	1.31 (1.02-1.67)	1.29 (1.00-1.66)	1.18 (0.91-1.52)
	Frequently	371	27	1.90 (1.29-2.78)	1.82 (1.24-2.67)	1.75 (1.19-2.57)	1.47 (1.00-2.17)
				p_{trend} <0.0001	p_{trend} =0.0005	p_{trend} =0.038	p_{trend} =0.031
**Antidepressant medication**							
**SLOSH**	Never	8979	200	*Ref*	*Ref*	*Ref*	*Ref*
	Occasionally	425	15	1.59 (0.94-2.68)	1.49 (0.88-2.52)	1.48 (0.87-2.50)	1.36 (0.80-2.31)
	Frequently	76	4	2.39 (0.89-6.43)	1.94 (0.72-5.23)	1.93 (0.71-5.21)	1.58 (0.58-4.30)
**WEHD**	Never	14840	542	*Ref*	*Ref*	*Ref*	*Ref*
	Occasionally	694	31	1.69 (1.03-2.77)	1.61 (0.98-2.64)	1.58 (0.97-2.60)	1.36 (0.83-2.24)
	Frequently	295	22	3.23 (1.94-5.71)	3.07 (1.78-5.28)	2.98 (1.73-5.15)	2.29 (1.32-3.97)
**Pooled**	Never	23819	742	*Ref*	*Ref*	*Ref*	*Ref*
	Occasionally	1119	46	1.64 (1.15-2.35)	1.55 (1.08-2.23)	1.53 (1.07-2.20)	1.36 (0.95-1.95)
	Frequently	371	26	3.01 (1.88-4.84)	2.76 (1.71-4.45)	2.70 (1.67-4.35)	2.10 (1.30-3.40)
				p_{trend} <0.0001	p_{trend} <0.0001	p_{trend} =0.0015	p_{trend} =0.0009

Model 1 adjusted for: sex, age, marital status, cohabiting with children, education and baseline year. Model 2 adjusted for covariates in model 1+T1 job demands and T1 decision authority. Model 3 adjusted for covariates in model 2+T1 depressive symptoms. HR=Hazard rate, CI=confidence interval.

Supplementary table 6. Estimated E-values.

Outcome	E-value[1] (point estimate)	E-value[1] (lower limit of CI)
Psychotropic medications	1.58	1
Antidepressants	2.05	1.44

[1] E-value are based on pooled meta-analytic estimates using model 3 (adjusted for sex, age, marital status, child cohabitation, baseline year and baseline levels of depressive symptoms, job demands and decision authority)

The e-value is a quantification of how strongly related any unmeasured confounder(s) would need to be with the exposure and the outcome, in order to nullify our results (i.e. retrieve a HR of 1 and/or have a 95% confidence interval including 1). For example, in the examined association between onset of workplace bullying and incident antidepressant use, any unmeasured confounder(s) would need to be associated with a 2.05 times higher risk (after adjustment for covariates used in model 3) of both onset of workplace bullying and incident treatment with antidepressants to shift our estimated HR to 1. Likewise, it would need to be associated with a 1.4 times higher risk of the exposure and the outcome to make our 95% CI include unity.

Supplementary figure 7. Subgroup analyses of the association between workplace bullying and incident treatment with psychotropic medication/antidepressants during 2 years of follow-up starting at T2.

**All psychotropic medication**	**N**	**N exposed**	**N cases**	**HR (95% CI)**
SLOSH	9480	501	489	1.28 (0.92-1.79)
WEHD	15829	989	595	1.20 (0.91-1.60)
Women	13363	901	706	1.26 (0.97-1.63)
Men	11946	589	378	1.20 (0.80-1.82)
Without elevated depressive symptoms at T1	24416	1373	1008	1.18 (0.93-1.49)
Employed at same organization T1-T2*	7603	368	392	1.25 (0.85-1.85)
Changed employer T1-T2*	1877	133	97	1.40 (0.74-2.65)
**Antidepressants only**				
SLOSH	9480	501	219	1.40 (0.87-2.26)
WEHD	15829	989	246	1.66 (1.13-2.43)
Without elevated depressive symptoms at T1	24416	1373	420	1.51 (1.09-2.09)

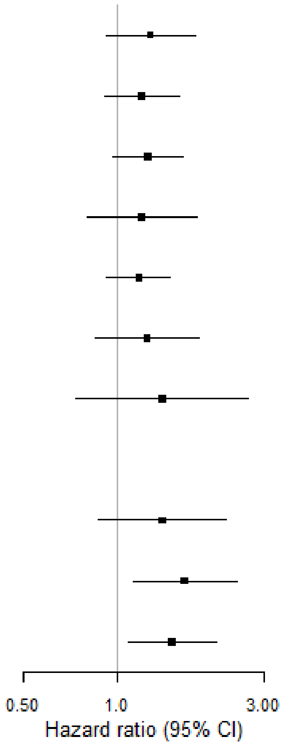

Estimates are derived from the most adjusted models. HR=Hazard rate, CI=confidence interval.
*=analysis only performed in SLOSH

Supplementary figure 8. Flowchart of sample selection process for sensitivity analyses, starting follow-up 6/12 months earlier.

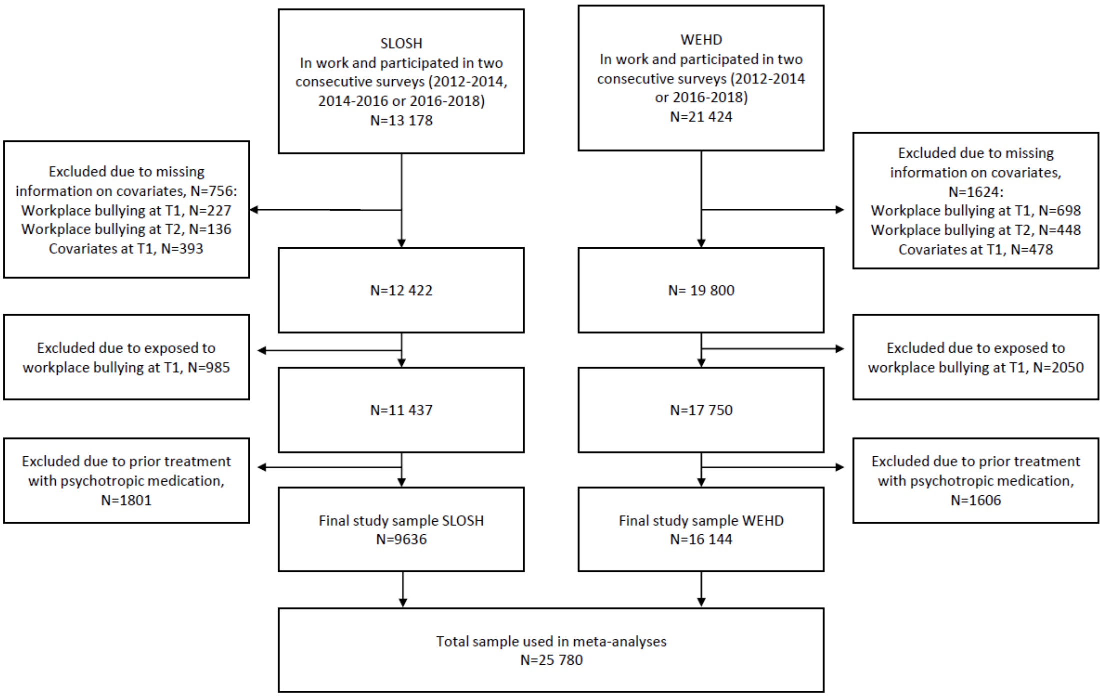

Supplementary table 7. Characteristics of the study samples used for sensitivity analysis, starting follow-up 6/12 months earlier.

	**SLOSH N=9636**	**WEHD N=16144**
Sex		
Male	4400 (45.7)	7704 (47.7)
Female	5236 (54.3)	8440 (52.3)
Age	49.6 (9.6)	46.1 (10.4)
Marital status		
Single	1831 (19.0)	3162 (19.6)
Married/cohabiting	7805 (81.0)	12982 (80.4)
Cohabiting w children		
No	5407 (56.1)	8134 (50.4)
Yes	4229 (43.9)	8010 (49.6)
Education		
Low	4502 (46.7)	8923 (55.3)
Intermediate	737 (7.6)	4583 (28.4)
High	4397 (45.6)	2638 (16.3)
Depression score[1]	4.3 (4.4)	7.0 (6.4)
Demands score[2]	7.5 (1.7)	8.9 (1.7)
Decision authority score[3]	6.3 (1.4)	7.6 (0.9)
Baseline year		
2012	3589 (37.2)	8023 (49.7)
2014	4937 (51.2)	-
2016	1110 (11.5)	8121 (50.3)
Onset workplace bullying		
Yes	512 (5.3)	1027 (6.4)
No	9124 (94.7)	15117 (93.6)
Frequency workplace bullying		
Never	9124 (94.7)	15177 (93.6)
Sometimes	434 (4.5)	722 (4.5)
≥Monthly	78 (0.8)	305 (1.9)

Presented as N (%) or mean (SD).

[1]range 0-24 (SLOSH)/0-50 (WEHD)

[2]range 3-12 in both cohorts

[3]range 2-8 in both cohorts

Supplementary figure 9. Association between workplace bullying and incident treatment with psychotropic medication including cases that occurred between T1 and T2.

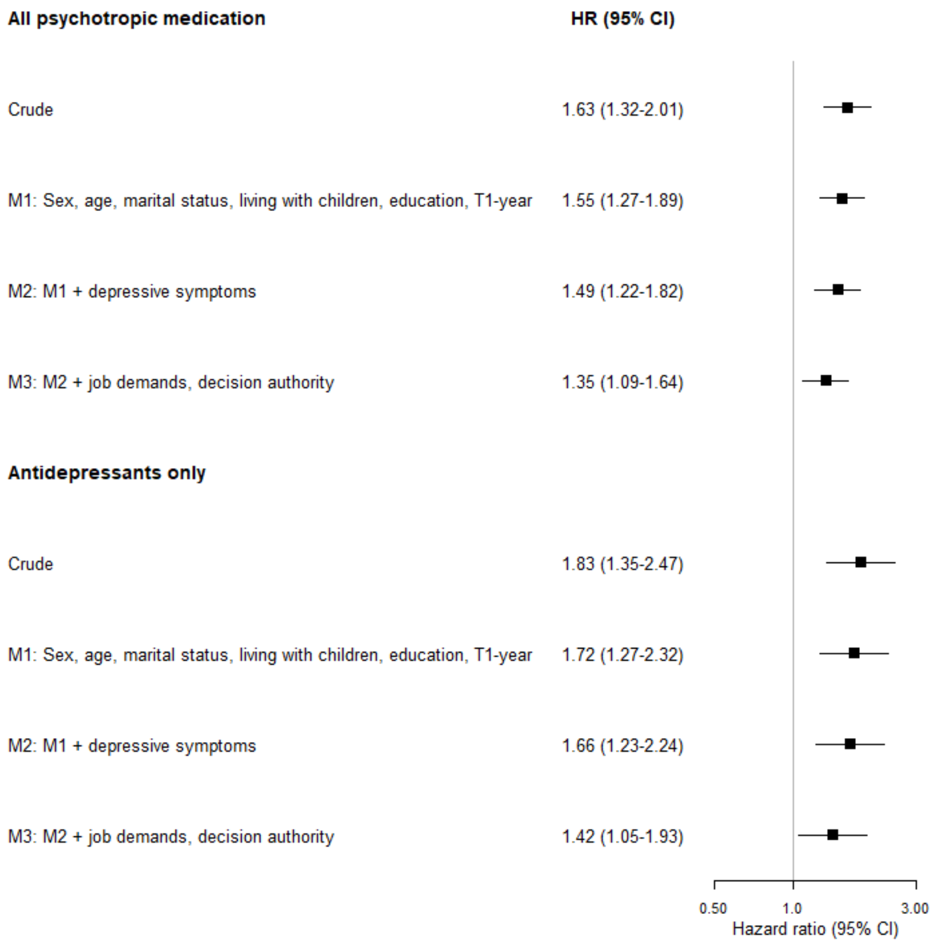

Supplementary table 8. Cohort-specific and pooled estimates from sensitivity analysis using Cox proportional hazards regressions, examining the association between onset of workplace bullying and incident treatment with psychotropic medications/antidepressants during 2 years of follow-up starting at the exposure reference period (T2 minus 6 months in SLOSH, T2 minus 12 months in WEHD).

			SLOSH				WEHD				Pooled estimate
**Outcome**	**Subgroup**	**Model**	**N**	**N exp**	**N cases**	**HR (95% CI)**	**N**	**N exp**	**N cases**	**HR (95% CI)**	**HR (95% CI)**
Psychotropic medication	All	Crude	9636	512	532	1.50 (1.09-2.07)	16144	1027	628	1.73 (1.34-2.32)	1.63 (1.32-2.01)
		Model 1				1.39 (1.01-1.92)				1.66 (1.28-2.14)	1.55 (1.27-1.89)
		Model 2				1.32 (0.96-1.83)				1.61 (1.25-2.09)	1.49 (1.22-1.82)
		Model 3				1.22 (0.88-1.68)				1.42 (1.09-1.84)	1.35 (1.09-1.64)
	Women	Crude	5236	326	376	1.57 (1.11-2.23)	8440	606	396	1.34 (0.96-1.88)	1.45 (1.14-1.84)
		Model 1				1.54 (1.08-2.18)				1.33 (0.95-1.87)	1.43 (1.12-1.82)
		Model 2				1.46 (1.03-2.08)				1.30 (0.93-1.83)	1.37 (1.08-1.75)
		Model 3				1.38 (0.97-1.96)				1.14 (0.81-1.61)	1.25 (0.98-1.60)
	Men	Crude	4400	186	156	0.90 (0.40-2.03)	7704	421	232	2.44 (1.64-3.62)	2.01 (1.41-2.88)
		Model 1				0.91 (0.40-2.05)				2.43 (1.64-3.62)	2.02 (1.41-2.88)
		Model 2				0.86 (0.38-1.95)				2.35 (1.57-3.50)	1.93 (1.35-2.77)
		Model 3				0.75 (0.33-1.69)				2.08 (1.39-3.11)	1.70 (1.19-2.44)
Antidepressant medication	All	Crude	9636	512	239	1.28 (0.77-2.13)	16144	1027	247	2.21 (1.52-3.20)	1.83 (1.35-2.47)
		Model 1				1.16 (0.70-1.93)				2.12 (1.46-3.08)	1.72 (1.27-2.32)
		Model 2				1.10 (0.66-1.83)				2.07 (1.43-3.02)	1.66 (1.23-2.24)
		Model 3				0.99 (0.59-1.65)				1.73 (1.18-2.52)	1.42 (1.05-1.93)

SLOSH=Swedish Longitudinal Occupational Survey of Health. WEHD=Work Environment and Health in Denmark Study, HR=Hazard rate, CI=confidence interval. Model 1 adjusted for: sex (not in sex-stratified analyses), age, marital status, cohabiting with children, education and baseline year. Model 2 adjusted for covariates in model 1+T1 job demands and T1 decision authority. Model 3 adjusted for covariates in model 2+T1 depressive symptoms.
